# Attentional bias for alcohol-related cues in a clinical setting among patients with alcohol use disorder: Evidence from eye movements and reaction times

**DOI:** 10.1016/j.dadr.2026.100438

**Published:** 2026-04-15

**Authors:** Isabella Fuchs-Leitner, Nikolas Gerstgrasser, Kurosch Yazdi-Zorn, Harald Kindermann, Jan Rosenleitner

**Affiliations:** a Department of Psychiatry – Specialization Addiction Medicine, Kepler University Hospital, Linz, Austria; b Faculty of Medicine, Johannes Kepler University Linz, Austria; c Department of Marketing and Electronic Business, University of Applied Sciences Upper Austria, Steyr, Austria

**Keywords:** Alcohol Use Disorder, Attentional Bias, Eye-tracking, Reliability, Craving

## Abstract

**Background:**

Alcohol-related attentional bias (AB) is a central mechanism in alcohol use disorder (AUD), yet its temporal dynamics and measurement reliability remain debated. This study investigated the psychometric properties and temporal characteristics of AB in a clinical sample using a multimodal approach.

**Methods:**

Patients with AUD (N = 49) completed a visual-probe task at short (200 ms) and long (2000 ms) stimulus-onset asynchronies (SOAs). Reaction times (RT) and eye-tracking (ET) indices were evaluated, including dwell time, fixation count, and first fixation landing position, with internal reliability assessed specifically for AB indices (difference scores) and correlations across modalities.

**Results:**

RT-based AB-indices showed moderate split-half reliability at short and good reliability at long SOAs. ET-base AB indices of dwell time and fixation count demonstrated good reliability. Contrary to some prior studies, multimodal measures were interrelated, with significant correlations between dwell time and RT-based AB indices at both SOAs. RTs revealed a biphasic pattern via a significant approach bias at 200 ms and avoidance at 2000 ms. However, ET indices showed no significant effects for dwell time or fixation count. AB correlated with momentary craving, which was generally low in this clinical sample, but not with AUD severity. No significant differences were observed based on alcohol type preference.

**Conclusions:**

Manual RTs captured the dynamic pattern of the approach-avoidance time course of AB. Findings support a state-dependent perspective, suggesting AB reflects a fluid motivational state rather than a stable trait. Overall, the results indicate that AB indices can be measured with acceptable reliability, supporting further investigation of attentional processes in AUD.

## Highlights

• Visual-probe task shows moderate-to-good reliability for reaction times.
• Dwell time and fixation count show good reliability in eye tracking.
• Alcohol-related bias follows a biphasic approach-avoidance pattern.
• Attentional bias in AUD reflects a dynamic state influenced by craving.

## 1 Introduction

### 1.1 Attentional bias in addiction

Visual attention serves to prioritize relevant information over irrelevant, supporting efficient cognitive processing and continuous sensory input (Corbetta and Shulman, 2002, Egeth and Yantis, 1997). Attention can be deployed overtly, through eye movements that align gaze with the attended stimulus, or covertly, when attention shifts without corresponding ocular motion (Carrasco, 2011, Posner, 1980). In addictive disorders, this prioritization is often observed as substance-related attentional bias (AB), characterized by the preferential allocation of attentional resources toward substance-related stimuli (Anderson, 2016, Field and Cox, 2008). Within alcohol use disorder (AUD), this bias is directed specifically toward alcohol-related cues. The incentive-sensitization theory suggests that repeated exposure to alcohol-associated stimuli increases the sensitivity of the brain’s reward system, rendering these cues especially salient and attention-grabbing (Berridge and Robinson, 2016, Robinson and Berridge, 2008). Expanding upon this, the state vs. trait framework proposed by Field et al. (2016) suggests that AB fluctuates depending on momentary appetitive and aversive evaluations rather than reflecting a stable trait. In clinical AUD samples, attentional responses to alcohol cues vary dynamically with contextual and motivational factors (Bollen et al., 2024, Ghiţă et al., 2024). AB is shaped by cue salience, motivational state, and individual differences, resulting in dynamic and context-dependent attentional allocation (Bollen et al., 2022, Field et al., 2016).

### 1.2 Temporal dynamics of attentional bias

Behavioral patterns in clinical AUD samples are complex, often showing a dynamic approach-avoidance pattern, where initial attentional capture (approach) towards alcohol cues is followed by rapid disengagement or voluntary avoidance (Beraha et al., 2018, Noël et al., 2006). Similar approach-avoidance patterns in both clinical and healthy control participants suggest that these dynamics may not be specific to AUD (Vollstadt-Klein et al., 2009). To capture these temporal aspects of AB, experimental paradigms often manipulate stimulus-onset asynchronies (SOAs), defined as interval between cue presentation and target appearance. Short SOAs (approximately 50–200 ms) are typically used to capture initial orienting, while longer SOAs (>1000 ms) allow evaluation of later processes such as disengagement or sustained attention. Exact temporal dynamics, however, remain unclear. For instance, Townshend and Duka (2007) found avoidance bias in recently detoxified patients with an SOA of 500 ms. Hence, further examining different SOAs helps characterize the dynamic attentional responses to alcohol cues in AUD samples (Bollen et al., 2024, Noël et al., 2006).

### 1.3 Methodological considerations in AB assessment

Traditional reaction time (RT) tasks, such as the dot-probe task, remain standard tools due to their ease of implementation and ability to provide a summary measure of the final motor output of processing (Cox et al., 2014). According to the premotor theory of attention, spatial attention and motor preparation are tightly coupled, and each overt eye movement is preceded by a covert shift of attention (Rizzolatti et al., 1987, Smith and Schenk, 2012). While RT-based indices might provide insight into early orienting processes that may not be captured by physical gaze patterns alone (Klonteig et al., 2025, Waechter et al., 2014), they are indirect measures of visual attention and often show low internal reliability (Ataya et al., 2012, Christiansen et al., 2015a, Jones et al., 2018). In clinical AUD samples, RT-based AB measures tend to exhibit particularly low consistency, likely due to motor execution noise, response variability, and small effect sizes of attentional bias (Bollen et al., 2025). However, recent work suggests that alternative computational approaches can improve the reliability of RT-based indices (Carlson and Fang, 2020, Evans and Britton, 2018).

To complement these behavioral measures, researchers use eye-tracking, which offers a direct and continuous record of overt visual attention with high temporal and spatial resolution (Maurage et al., 2020). Eye tracking adds nuance, distinguishing between early and later stages of attention, providing insights into the dynamic time course of bias (Roberts and Fillmore, 2015). Recent studies applying eye tracking in alcohol cue paradigms have further characterized the temporal dynamics of attentional allocation toward alcohol stimuli (Bollen et al., 2024, Ghiţă et al., 2024). Eye tracking metrics, such as dwell time and fixation count, can demonstrate higher internal reliability compared to RT indices (e.g., Cronbach’s α ≥0.90 in free‑viewing tasks; Bollen et al., 2023; Soleymani et al., 2020). However, eye tracking is also subject to limitations including pupil detection failures or occlusion, potentially resulting in high rates of data loss (Holmqvist et al., 2012). Combining RT and eye tracking allows for a more comprehensive and robust understanding of how attention is allocated towards alcohol-related stimuli (Bollen et al., 2021b, Christiansen et al., 2015a, Field and Cox, 2008). Studies including clinical AUD samples suggest that eye-tracking indices in VPT paradigms may provide higher reliability than RT-based measures (Bollen et al., 2021b, Bollen et al., 2025).

### 1.4 AB in clinical populations and clinical implications

Despite strong theoretical accounts of AB, empirical findings remain inconsistent, particularly in clinical populations. A systematic review by Bollen et al. (2022) reported that AB is frequently observed in subclinical samples but often absent or weak in individuals with AUD. These discrepancies may reflect both the dynamic nature of attentional responses and methodological differences across studies, including task design, stimulus timing, and individual differences in craving or AUD severity (Bollen et al., 2022). Understanding these temporal dynamics is important since AB may contribute to the maintenance of addiction (Cox et al., 2002), although a recent review reported inconsistent findings concerning the relationship between AB and recurrence of alcohol use (Topbaş et al., 2025).

Attentional Bias Modification (ABM) aims to retrain attentional patterns (Cox et al., 2014), but findings are mixed and ambiguous (Boffo et al., 2019, Christiansen et al., 2015b). While some trials report modest increases in abstinence following training (Manning et al., 2021, Manning et al., 2016), recent meta-analyses indicate small or null effects on both bias and clinical outcomes (Cristea et al., 2016, Pan et al., 2026), highlighting the need for improved task sensitivity and multimodal assessment, especially in clinical AUD samples (Heitmann et al., 2018).

### 1.5 The present study

The present study further investigates alcohol-related AB in a clinical sample of patients with AUD. As this study was not pre-registered, it should be considered exploratory (Bollen et al., 2024). RT and eye-tracking measures were included to capture potentially dissociable covert and overt components of attention, and to assess the reliability of AB indices for both modalities. Stimuli were salience-adjusted and never repeated to minimize perceptual confounds and habituation effects. RTs were used to examine potential approach-avoidance patterns at short and long SOAs, while eye-tracking was analyzed for long SOA trials only, comparing alcohol and non-alcohol AOIs. The stimulus set included three beverage categories, allowing exploratory investigation of personalized cue effects (alcohol type preference). Finally, we analyzed correlations between AB, momentary craving, and AUD severity to explore potential state- versus trait-like influences.

## 2 Methods

### 2.1 Participants

#### 2.1.1 A-priori sample size calculation

An a-priori power analysis was conducted using G*Power (Faul et al., 2007) for a repeated-measures ANOVA with four measurements (corresponding to the cells of the planned 2 ×2 within-subjects design for RTs). Assuming a small-to-medium interaction effect (Cohen’s *f* = 0.20) and standard settings (alpha =.05, power =.80, correlation among repeated measures =.50), the required sample size was 36 participants. Our final sample consisted of 49 participants, providing adequate power to detect the expected interaction and main effects, as well as sufficient power for planned paired-sample *t*-tests (eye movement indices).

#### 2.1.2 Inclusion and exclusion criteria

A total of 49 inpatients (16 identified as females, 33 as males; age 18–64 years, *M* = 45.3 years) who had completed acute detoxification and were receiving inpatient treatment for AUD at the Kepler University Hospital were included in the analysis of this study. Participants were required to be fluent in German. Exclusion criteria included contraindications for eye tracking (e.g., severely impaired vision or color vision, ophthalmic or neurological disorders), polysubstance use, and neurological diseases. Of the originally recruited 57 patients, seven were excluded before analysis due to tremor, cataract, impaired hearing, or inability to comprehend the instructions, and one additional participant was excluded due to poor eye-tracking calibration (N = 47). The study was conducted in accordance with the Declaration of Helsinki and approved by the local ethics committee. Patients participated on a voluntary basis, and written informed consent was obtained by all participants.

### 2.2 Apparatus and stimuli

All sessions were conducted individually in a quiet, dimly lit room with standardized lighting. Participants first completed interview-administered questionnaires. They then completed a computerized task while seated at a workstation with an adjustable chair and a chin- and forehead rest, positioned 65 cm away from a 22-inch monitor (1680 × 1050 px, 60 Hz). Responses in the Visual Probe Task were made using the “X” key (left index finger) and the “comma” key (right index finger), both marked for clarity. Eye movements were recorded with a screen-based eye tracker (SMI RED, SensoMotoric Instruments) at 120 Hz using a validated 9-point calibration. Each session lasted approximately 30 min.

#### 2.2.1 Stimulus set

**Fig. 1:**
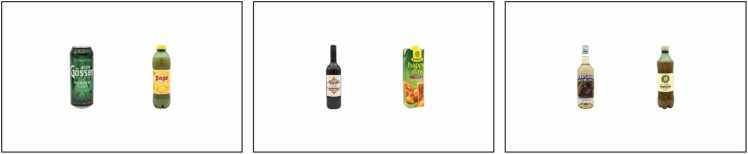
Stimuli. Examples of stimulus pairs depicting one alcoholic and one non-alcoholic beverage each. Alcoholic beverages belonged to one of three categories: beer (left panel), wine (middle panel), and spirits or liquors (right panel).

To elicit robust attentional effects while minimizing potential confounds, stimuli were designed controlling for visual saliency, presentation order, and memory effects while preserving the natural appearance of familiar beverages. This was considered crucial for ecological validity. Custom photographs of Austrian-brand bottles, cans, and cartons were used, with backgrounds removed and images standardized in size and presentation. The final stimulus set included 96 image pairs (plus six practice pairs), each showing one alcoholic and one non-alcoholic beverage on a white background. Pairs were evenly distributed across three categories (beer, wine, liquor; 32 pairs each; see Fig. 1). Images measured 308 × 461 pixels (8.66 × 13 cm) with a 7 cm horizontal gap, and left-right positioning was fully counterbalanced.

To control for visual saliency, we applied the computational saliency model proposed by Itti et al. (1998) using the MATLAB Saliency Toolbox (Walther and Koch, 2006). The model was used to predict the most salient point in each image, corresponding to the likely first fixation location. Stimulus pairs were iteratively rearranged and evaluated until the final set met the following criteria: the most salient area appeared equally often in alcoholic and non-alcoholic beverage images and was evenly distributed across both screen positions. Stimuli were organized into four subsets corresponding to the four experimental conditions (short congruent, short incongruent, long congruent, long incongruent), each balanced in regards to visual salience, screen side, and beverage category (beer, wine, liquor). Presentation order was randomized individually for each participant.

### 2.3 Procedure

#### 2.3.1 Questionnaires

Participants completed several standardized questionnaires, which provided complementary information on dependence severity, consumption, and craving:

• Severity of Dependence Scale (SDS): 5-items assessing psychological dependence, Cronbach’s α ≈ .83 (Gossop et al., 1995). In the present sample, internal consistency was acceptable (α =.68).
• Alcohol Use Disorders Identification Test (AUDIT): 10 items evaluating alcohol use, Cronbach’s α ≈ .80–.85 (German Version: Dybek et al., 2006; Saunders et al., 1993). Internal consistency in the present sample was α = .63.
• Alcohol Craving Questionnaire - Short Form Revised (ACQ-SF-R): 12 items covering craving intensity, expected affect, and perceived control; Cronbach’s α ≈ .85–.93 (Rodrigues et al., 2021, Singleton, 1995). Internal consistency in the present sample was excellent (α =.93).
• Visual Analogue Scale (VAS) for craving: 10-point Likert scale on which participants rated current craving intensity from 0 (“not at all”) to 100 (“very strong”).

#### 2.3.2 Visual probe task (VPT)

The main task used a visual probe paradigm to assess attentional shifts. Each trial began with a 500 ms fixation check on a central cross, and insufficient fixation triggered recalibration and trial restart. Successful fixation was confirmed by a drift check, after which the fixation cross disappeared. The stimulus pair was then presented for 200 ms (short SOA) or 2000 ms (long SOA), followed by a target probe - a grey dot. Participants were asked to indicate the position of the target probe (left or right) as quickly as possible within 3000 ms. A blank screen of 500–1500 ms preceded the next trial. Trial order was randomized, and eye movements and reaction times were recorded. The short SOA was chosen to capture early attentional orienting (approach) in RTs, whereas the long SOA was intended to capture potential disengagement (avoidance) in RTs while allowing sufficient time for eye movements to occur. The task included 96 trials preceded by six practice trials. The experiment was designed to run continuously, although participants could take individual breaks if needed.

#### 2.3.3 Data processing

Incorrect responses were excluded from further analysis (0.8%). For reaction times, mean RTs were first calculated separately for each condition: congruent (target on same side as alcohol image) and incongruent (target on opposite side), as well as for short (200 ms) and long SOAs (2000 ms), and outliers (± 2 SD from condition means) were omitted (4.2%). Subsequently, AB indices were computed as the difference between incongruent and congruent RTs for each SOA. Descriptive statistics revealed some negative skew (short SOA: skew = −0.96; long SOA: skew = −1.81; kurtosis = 2.47–6.28), with Shapiro-Wilk tests indicating deviations from normality. Given the sample size, parametric analyses were considered robust.

For eye tracking metrics only long SOA trials were analyzed, as the short SOA was insufficient to yield meaningful eye-movement measures. Two participants were excluded due to a tendency to fixate the screen center (N = 47). Fixations shorter than 80 ms or longer than 1000 ms (1.4% of data) were excluded. For subsequent analysis, two areas of interest (AOIs) were defined around the alcoholic and non-alcoholic images, each matching the image size (308 × 461 pixels). For each trial, fixation data were aggregated separately within each AOI.

Eye-tracking metrics were selected a priori to capture distinct aspects of alcohol-related AB: dwell time (total fixation duration) and fixation count (number of fixations) reflect sustained attention, fixation-duration measures index depth of processing (Rayner et al., 2007), and latency measures capture attentional timing (*mean AOI latency)* and initial orienting (*mean AOI first latency*), allowing assessment of immediate and maintained AB. AB indices for eye-tracking were calculated as the difference between alcohol and non-alcohol AOIs. Descriptive statistics indicated generally near-normal distributions for eye-tracking AB indices (skews for: dwell time = 0.49; fixation count = 0.34; fixation duration = −0.13; first fixation duration = −1.05; mean fixation latency = 1.01; first fixation latency = −0.15), with Kolmogorov–Smirnov and Shapiro–Wilk tests confirming these patterns. Distributions were considered suitable for parametric analyses, and AB indices remained the primary measures of alcohol-related AB.

### 2.4 Statistical analyses

All analyses were conducted using SPSS 28.0. First, reliability was calculated for the AB indices for both RT and eye-tracking measures. Split-half correlations were computed by dividing trials into two fixed stimulus sets that included all experimental conditions (SOA: long/short; congruency: congruent/incongruent; alcohol stimulus position: left/right; and the three beverage types). As trials were presented in randomized order, no systematic order effects were expected. Pearson correlations between the two halves were corrected using the Spearman–Brown formula. Cronbach’s alpha was not calculated because it is not appropriate for difference scores (Evans and Britton, 2018). Only AB indices with adequate split-half reliability were included in further analyses.

Second, AB effects were evaluated via ANOVA and pairwise comparisons. Reaction times were analyzed using a 2 × 2 repeated-measures ANOVA with the factors congruency (congruent vs. incongruent) and interval (short vs. long SOA). Bonferroni-adjusted *t*-tests were applied for post-hoc comparisons of the significant RT interaction (four pairwise comparisons; reported p-values are Bonferroni-adjusted). For Eye-tracking metrics, participant-level AOI averages were compared using paired-sample t-tests. Effect sizes in the ANOVA are expressed as *η²* (eta squared), Cohen’s *d* is reported for *t*-tests.

Third, AB indices were calculated separately for alcohol type (beer, wine, and liquor). Participants reported which types they preferred and regularly consumed, and multiple endorsements were allowed if no single type was clearly preferred. For each alcohol type, participants were classified as preferring or not preferring it, and pairwise *t*-tests were used to compare AB between these groups.

Finally, Spearman correlations were computed to examine relationships between RT and eye-tracking AB indices, as well as AUD severity (AUDIT, SDS) or craving (ACQ-SF-R, VAS).

## 3 Results

### 3.1 Reliability of AB indices

All results are displayed in Table 1. For RTs, the split-half reliability for the AB index was moderate in short SOAs (*r*_SB_ =.60) and good in long SOAs (*r*_SB_ =.74).

**Table 1:** Split-half reliability of attentional bias (AB) indices.

**Variable**	**Description**	***r***	***p***	***r***_**SB**_	
RT – short SOA	RT AB in short SOA (200 ms)	.429	.002	.600	Moderate
RT – long SOA	RT AB in long SOA (2000 ms)	.586	< .001	.739	Good
Dwell Time	Total duration of all fixations within an AOI	.624	< .001	.768	Good
Fixation Count	Number of fixations occurring in the AOI per trial	.632	< .001	.774	Good
Fixation Duration	Average duration of fixations within the AOI	.315	.031	.479	Moderate
First Fixation Duration	Duration of the first fixation entering the AOI	.190	.201	.319	Low / not significant
AOI Latency	Average timing of attention across all fixations in the AOI	.273	.063	.429	Low / not significant
AOI First Latency	Latency of the first fixation entering the AOI	-.106	.478	-.237	Unreliable

*Note*. RT =  reaction time, AB =  attentional bias, SOA =  stimulus onset asynchrony, AOI =  area of interest. AB indices were calculated for RTs (i.e, difference between incongruent and congruent condition) for the short and long SOA. Eye tracking AB indices were computed only for the long SOA, based on the AOI content (i.e., difference between alcohol and non-alcohol). Split-half reliability was assessed using Pearson correlations (original r and *p*-values) corrected with the Spearman-Brown formula (*r*_SB_).

For eye tracking AB indices, results indicate good reliability for dwell time (*r*_SB_ =.77) and fixation count (*r*_SB_ =.77), and moderate reliability for mean fixation duration (*r*_SB_ =.48). All other indices failed to reach significance and sufficient reliability. Only the three significant variables demonstrating sufficient reliability were included in subsequent analyses.

### 3.2 AB effects: pairwise comparisons

**Fig. 2:**
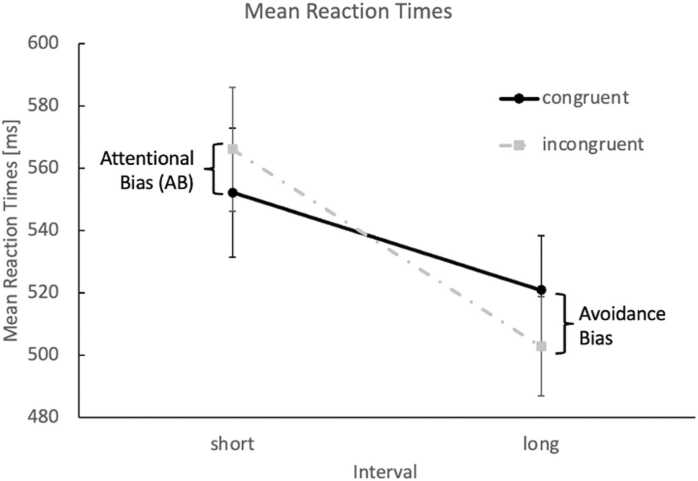
Reaction Time Results. Displayed are the mean reaction times (RTs; error bars represent SEM) for congruent trials (alcohol and dot probe on the same side; black solid line) and incongruent trials (gray dashed line), shown separately for the short interval (200 ms, left) and the long interval (2000 ms, right). RT patterns indicate an attentional bias in the short and an avoidance bias in the long interval.

For RTs, a 2 × 2 repeated-measurements ANOVA revealed a significant main effect for interval, *F*(1, 48) = 18.72, *p* < .001, *η²* = .281, with generally faster responses in the long (512 ms) compared to the short interval (559 ms). Crucially, a significant interaction between congruency and interval was found, *F*(1, 48) = 24.81, *p* < .001, *η²* = .341. Post-hoc Bonferroni adjusted *t*-tests revealed an AB for alcoholic beverages in the short interval (congruent = 552 ms, incongruent 566 ms, difference = 14 ms, *t*(48) = 2.44, *p*_Bonf_ = .018, *d* = 0.35). This effect reversed in the long interval (congruent = 521 ms, incongruent = 503 ms, difference = −18 ms; *t*(48) = 2.87, *p*_Bonf_ = .006, *d* = 0.41), indicating an avoidance bias away from alcohol (see Fig. 2).

Model assumptions were evaluated by inspecting standardized residuals of the repeated-measurements ANOVA. Q-Q plots indicated that residuals largely followed the expected normal distribution, with only minor deviations in the upper tail. To further assess robustness against skewness, the same ANOVA was repeated using log-transformed RTs (ln-transformation), which yielded an identical pattern of results including the significant main effect of interval and the significant congruency ×  interval interaction (both *p*s < .001).

**Fig. 3:**
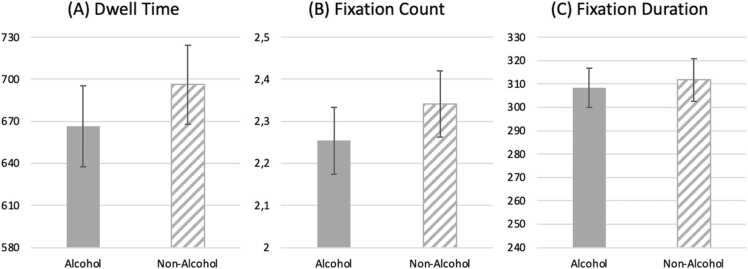
Eye tracking indices. Mean eye-movement measures for alcohol and non-alcohol areas of interest (AOIs). For each trial, fixation data were aggregated within each AOI and then averaged across trials for each participant. Bars depict means and error bars indicate the standard error of the mean (SEM). Panels show (A) dwell time (total AOI fixation duration in ms), (B) fixation count (number of fixations), and (C) mean fixation duration (in ms). Note that the y-axis scales differ across panels.

For eye tracking measures, we found that although non-alcohol AOIs were fixated longer (see panel A, Fig. 3), the difference was not significant (*dwell time*: alcoholic AOI mean = 667 ms, non-alcoholic mean = 696 ms; *t*(46) = 0.87, *p* = .388, *d* = 0.13). Fixation count and mean fixation duration did barely differ between the two AOIs (*fixation count*: alcoholic AOI mean = 2.25, non-alcoholic mean = 2.34; *t*(46) = 0.98, *p* = .331, *d* = 0.14; *mean fixation duration*: alcoholic AOI mean = 308 ms, non-alcoholic mean = 312 ms; *t*(46) = .48, *p* = .637, *d* = 0.07).

### 3.3 Alcohol type preference

Pairwise *t*-tests comparing patients reporting a preference versus no preference for beer, wine, or liquor (see Table 2) revealed no significant results for RTs (all *p*s > .13), nor for the eye tracking variables (all *p*s > .082). Since no significant results were observed, no further correction for multiple testing was applied. Nevertheless, several moderate numerical differences were observed. Patients who preferred beer had lower RT-based AB indices than those without a beer preference in the short SOA (*M*: yes = 12 ms vs. no = 37 ms, *d* = 0.47), and stronger negative AB indices in the long SOA (*M*:_yes = −28 ms vs.no = −4 ms, *d* = 0.44), indicating stronger avoidance for those who preferred beer. For wine, patients who preferred it showed a negative AB in dwell times compared to non-preferring patients (*M*: yes = −77 ms vs. no = 26 ms, *d* = 0.42). Patients who preferred liquor tended to show higher RT-based AB indices in the short SOA (*M*: yes = 27 ms vs. no = 4 ms, *d* = 0.37) and higher dwell times (*M*: yes = 38 ms vs. no = −66 ms, *d* = 0.40), consistent with greater AB toward preferred liquor cues. Detailed results can be found in Table 2.

**Table 2:** Attentional Bias (AB) indices for preference of alcohol type and interval. Results of comparisons between patients with and without preference for the respective alcohol type.

**Alcohol Type**	**AB Index**	**Preference**	**N**	**Mean**	***t***	***p***	**Cohen’s*****d***
Beer	RT short SOA	No	17	36.6	1.6	.13	0.47
		Yes	32	11.6			
	RT long SOA	No	17	-4.4	1.5	.15	0.44
		Yes	32	-27.9			
	ET dwell time	No	16	-109.2	1.3	.19	0.40
		Yes	31	10.5			
	ET fixation count	No	16	-0.15	1.4	.18	0.42
		Yes	31	0.19			
	ET fixation duration	No	16	-42.9	1.8	.08	0.55
		Yes	31	-7.8			
Wine	RT short SOA	No	27	17.0	0.8	.41	0.24
		Yes	22	4.6			
	RT long SOA	No	27	-11.5	0.6	.55	0.17
		Yes	22	-20.8			
	ET dwell time	No	26	26.1	1.4	.16	0.42
		Yes	21	-76.8			
	ET fixation count	No	26	-0.01	1.3	.20	0.38
		Yes	21	-0.28			
	ET fixation duration	No	26	22.4	1.5	.13	0.45
		Yes	21	-11.1			
Liquor	RT short SOA	No	35	3.6	-1.2	.25	-0.37
		Yes	14	27.2			
	RT long SOA	No	35	-19.8	0.3	.78	-0.09
		Yes	14	-14.8			
	ET dwell time	No	35	-66.3	1.2	.24	0.40
		Yes	12	37.6			
	ET fixation count	No	35	-0.27	1.1	.28	0.37
		Yes	12	0.02			
	ET fixation duration	No	35	-0.1	0.0	.99	0.00
		Yes	12	0.0			

*Note*. AB index =  attentional bias index; RT =  reaction time; SOA =  stimulus onset asynchrony; short SOA (= 200 ms); long SOA (= 2000 ms); AOI =  area of interest. RT ABs (incongruent - congruent trials), ET AB (AOI_non-alcohol - AOI_alcohol). Reported *p*-values are two-sided. No results reached significance, and no correction for multiple testing was applied.

### 3.4 Craving, AUD severity, and correlational analyses

As expected, in this inpatient sample undergoing treatment for AUD, levels of alcohol consumption and dependence severity were high. Participants reported a mean score of *M* = 9.6 on the SDS (range 0–16), with scores ≥ 3 indicating alcohol dependence. On the AUDIT (range 0–40), the mean score was *M* = 26.9, well above the established cut-off values for harmful use (≥ 7 for women and ≥ 8 for men). In contrast, craving levels were relatively low. Mean craving indices on the ACQ-SF-R (total score divided by 12, range 1–7) were *M* = 1.9. Consistently, self-reported craving on the VAS (range 0–100) was low, with a mean score of *M* = 13.1. Concepts were significantly correlated for the two distinct craving measures (ACQ and VAS: *p* < .001), the two AUD severity scores (AUDIT and SDS: *p* < .001). Severity and craving were also related with the highest correlation between AUDIT and VAS Craving (*p* < .001). See Table 3 for full results (but please note that N = 47 due to ET indices.)

**Table 3:** Spearman correlations between AB indices, AUD severity, and craving (N = 47).

**Variable**	**1**	**2**	**3**	**4**	**5**	**6**	**7**	**8**
1. RT AB short SOA	--							
2. RT AB long SOA	.22	--						
3. AB Dwell Time	.31*	.32*	--					
4. AB Fixation Count	.22	.46**	.86***	--				
5. AB Fixation Duration	.33*	-.02	.62***	.22	--			
6. AUDIT (0−40)	-.17	.14	.07	.05	.06	--		
7. SDS (0−16)	-.11	.02	.16	-.03	.36*	.62***	--	
8. Craving ACQ-SF-R (1−7)	.25	.09	.36*	.33*	.20	.34*	.23	--
9. Craving VAS (0−100)	-.01	.32*	.15	.22	.07	.47***	.38**	.61***

*Note*. RT =  reaction time; AB =  attentional bias; SOA =  stimulus onset asynchrony; AUDIT =  Alcohol Use Disorders Identification Test; SDS =  Severity of Dependence Scale; ACQ-SF-R =  Alcohol Craving Questionnaire–Short Form–Revised; VAS =  visual analogue scale. Scale ranges are reported in parenthesis. Significance levels: *p < .05, **p < .01, ***p < .001.

Results are summarized in Table 3, and the main findings are discussed below. First, AB scores at the long SOA were significantly associated with AB indices derived from fixation duration (*ρ* =.46, *p* < .001) and dwell time (*ρ* =.32, *p* = .031). These findings suggest that AB measures with higher internal reliability were also interrelated. Craving assessed with the ACQ-SF-R was significantly correlated with both dwell time (*ρ* =.36, *p* = .012) and fixation count (*ρ* =.33, *p* = .026). Craving evaluated with the VAS was significantly correlated with RT-based AB scores in the long SOA (*ρ* =.32, *p* = .037). These results indicate that higher momentary craving is associated with greater attentional allocation toward alcohol cues. No significant correlation with AUDIT was observed at all.

## 4 Discussion

### 4.1 Reliability of AB measures

The present study investigated the psychometric properties and temporal characteristics of alcohol-related AB within a clinical sample of patients with AUD. We assessed reliability using split-half correlations and applied the Spearman-Brown formula for correction. A central finding of this study was the moderate-to-good reliability of RT-based AB indices, contrasting with prior reports characterizing the visual-probe task as having unacceptably low internal reliability (Ataya et al., 2012, Christiansen et al., 2015a). RT-based AB measures in the current study exhibited moderate split-half reliability for the short SOA and good reliability for the long SOA. The finding of increased reliability compared to earlier reports may be attributed to specific methodological choices, such as using salience-adjusted stimuli and avoiding repetition of stimuli to minimize habituation (Pennington et al., 2019), or to the clinical population with high AUD severity itself in contrast to other more heterogenous samples (Klonteig et al., 2025). Notably, our findings of high reliability for the RT-based AB index in the long SOA contrasts with recent reports by Bollen et al. (2021b), who reported low split-half reliabilities for RT indices in recently detoxified patients with severe AUD in a highly controlled study using the VPT. Although the overall design was similar to the current study, methodological and analytical details (e.g., background color, outlier handling, target probe, and stimulus repetition) and differences in population characteristics may explain this discrepancy. However, these contrasting findings in similar settings urge the need for further systematic investigation of the reliability of the VPT, especially in clinical populations.

In the current study, ET-based AB indices, specifically dwell time and fixation count, demonstrated satisfactory reliability, while mean fixation durations showed moderate reliability. These findings of high reliability align well with previous psychometric evaluations of eye tracking measures in alcohol-related AB although direct comparisons are limited since most studies used Cronbach’s alpha as reliability measure (Christiansen et al., 2015a, Soleymani et al., 2020). Studies using VPT in a clinical sample also report high reliability for ET indices (Bollen et al., 2021b, Bollen et al., 2025). Interestingly, despite the discrepancy observed for RT-based indices, the reliability of the dwell time AB index in the present study (r_SB_ ≈ 0.77) was comparable to that reported by Bollen et al. (2021), with reliability coefficients of approximately r_SB_ ≈ 0.76 and 0.80 for two different sessions.

Our multimodal AB measures were interrelated (e.g., by significant correlations between dwell time and RTs at both SOAs), in line with previous findings that eye-tracking captures the same underlying phenomenon as RT-based measures in some contexts (Christiansen et al., 2015a). However, this result contrasts with some prior research that found no such associations across modalities, leading some researchers to suggest that RT and eye-tracking may index distinct underlying phenomena or different processing stages (Klonteig et al., 2025, Price et al., 2015), with RT potentially serving only as an indirect measure contaminated by motor preparation and response execution noise (Bollen et al., 2021b). Conversely, other potential ET-based AB indices (e.g., mean first fixation duration and mean first AOI latency) exhibited poor reliability and were therefore excluded from further analyses. This pattern is consistent with prior evidence suggesting that early-stage capture indices often show lower or inconsistent psychometric reliability (Bollen et al., 2021b, Soleymani et al., 2020).

### 4.2 Temporal characteristics of AB

Regarding the temporal characteristics of AB, our results as measured by RTs revealed a distinct time course of AB, characterized by a significant approach bias at short SOAs (200 ms) and a significant avoidance bias at long SOAs (2000 ms). The observed biphasic pattern aligns well with prior research reporting similar approach-avoidance dynamics in clinical AUD samples. (Beraha et al., 2018, Noël et al., 2006, Vollstadt-Klein et al., 2009). In contrast, similar studies found no AUD-specific avoidance effect based on RTs in long SOAs (Bollen et al., 2021b, Bollen et al., 2025).

Notably, our ET results showed no significant effects for dwell time, fixation count, or mean fixation duration. This absence of a clear overt preference aligns with findings by Ghiţă et al. (2024), who reported no significant results for these variables when comparing patient with AUD and individuals reporting occasional alcohol use. Although our AB measures showed good reliability, the lack of clear eye-tracking effects indicates that reliable measurement does not necessarily imply (clinically) meaningful effects.

### 4.3 State-versus-trait theory in AB

Our findings further support the conceptualization of AB as a fluid state modulated by momentary craving rather than a stable trait of addiction severity (Bollen et al., 2024, Field et al., 2016). Although our sample exhibited high AUD severity, these measures (AUDIT, SDS) did not correlate with the magnitude of AB. Instead, significant correlations were found between AB and subjective craving, even though craving levels were generally low in the sample. In particular, higher craving was associated with greater attentional allocation toward alcohol cues in ET-based indices, indicating that participants reporting stronger craving tended to fixate longer and more often on the alcohol-related stimuli, whereas those with lower craving exhibited weaker AB. These findings are in line with recent reports that AB magnitude and direction (approach vs. avoidance) are primarily driven by temporary changes in appetitive and aversive evaluations of cues at the time of testing (Bollen et al., 2024, Field et al., 2016). Specifically, Bollen et al. (2024) observed using a free-viewing paradigm that severe AUD patients experiencing craving demonstrated an approach bias, while those without craving, which is common in recently detoxified inpatient settings, showed a stronger avoidance bias than healthy controls. This state-dependent perspective is also consistent with Christiansen et al. (2015b), who proposed that AB reflects an underlying motivational state linked to proximity to use, rather than a stable causal factor.

### 4.4 Effects of alcohol type preference

We found no significant difference between patients who reported a preference for a given alcohol type and those who did not, neither for RT- nor for ET-based AB indices. However, numerical differences and moderate effect sizes were observed. Attentional patterns varied across alcohol types, suggesting that attentional responses may differ depending on the preferred beverage. While personalized stimuli have been shown to increase reliability in non-clinical social drinkers (Christiansen et al., 2015a), the lack of effect in the current study may reflect insufficient number of trials to capture specific AB in a clinical population already prone to active avoidance (Ghiţă et al., 2024). Overall, these preliminary findings point to potential avenues for further exploration, but should be interpreted with caution given the small number of trials per alcohol type and the split sample according to preference.

### 4.5 Implications for research and interventions

Although alcohol-related AB has traditionally been proposed as a potential predictor of resumption of alcohol use (Cox et al., 2002), recent systematic evidence indicates that associations between AB and return to alcohol use are often inconsistent across substances (Topbaş et al., 2025). Moving forward, the clinical utility of AB assessment may benefit from approaches that account for the dynamic and state-dependent nature of attentional responses to alcohol cues. In the present study, AB was associated with momentary craving but not with AUD severity, supporting recent accounts that conceptualize AB as a fluctuating attentional state rather than a stable trait (Bollen et al., 2024). In addition, our multimodal design revealed partially divergent findings across RT and eye-tracking measures, suggesting that combining behavioral and gaze-based indices may provide a more comprehensive characterization of attentional processes (Ghiţă et al., 2024, Maurage et al., 2020). However, our findings also highlight important limitations for interpreting AB as a clinically meaningful intervention target (Christiansen et al., 2015b, Rodebaugh et al., 2016). Although both RT- and ET-based AB indices showed satisfactory reliability, reliable measurement alone does not necessarily imply clinical validity (Klonteig et al., 2025). Moreover, the absence of a healthy control group limits conclusions about the AUD-specificity of the observed effects, as the attentional patterns could reflect general appetitive salience rather than processes unique to AUD (Bollen et al., 2021a, Pennington et al., 2019).

### 4.6 Limitations

While this study adds to the extensive body of literature on alcohol-related AB (Field and Cox, 2008), several limitations should be considered. First, the generally low levels of subjective craving reported by patients may have limited the emergence of overt attentional preferences, as AB has been shown to be state dependent (Bollen et al., 2024, Christiansen et al., 2015b). Second, the absence of significant eye tracking effects despite high reliability of ET-based AB indices indicates that reliability alone does not guarantee validity or sensitivity for effect detection (Klonteig et al., 2025, Waechter et al., 2014). Third, the use of a standardized laboratory setting limits ecological validity relative to real-world drinking contexts (Pennington et al., 2019). Fourth, as this study was not pre-registered, the design and results should be considered exploratory (Bollen et al., 2024). Fifth, the lack of correlation between AB and AUD severity might reflect the homogeneity of the clinical sample, highlighting the need for future studies to include a broader range of consumption patterns, as the clinical specificity and long-term predictive value of AB remain debated (Topbaş et al., 2025). Finally, because stimuli were organized into fixed subsets corresponding to the experimental conditions, classical Cronbach’s alpha could not be computed at the single-trial level. This design limits comparability with prior studies and may reduce the generalizability of the reliability findings (Ataya et al., 2012).

### 4.7 Conclusions

In summary, the present study used a multimodal approach to examine alcohol-related AB in a clinical AUD sample. Both RT- and ET-based AB indices demonstrated moderate-to-high reliability based on split-half estimates corrected with the Spearman-Brown formula. RT measures revealed a temporal approach-avoidance pattern, with initial attentional capture by alcohol cues followed by avoidance at longer intervals. In contrast, ET measures such as dwell time and fixation count did not show significant AB effects. Together, these findings suggest that reliable measurement of AB does not necessarily correspond to consistent attentional effects across modalities. Consistent with recent theoretical accounts, our results also support the view that AB may reflect a dynamic attentional state influenced by momentary craving rather than a stable trait of addiction severity. However, given the absence of a control group and the lack of associations between AB and AUD severity, the clinical specificity and utility of the visual probe task for informing abstinence-focused interventions remain to be established.

## CRediT authorship contribution statement

**Isabella Fuchs-Leitner:** Writing – original draft, Project administration, Methodology, Investigation, Formal analysis, Data curation, Conceptualization. **Jan Rosenleitner:** Writing – review & editing, Validation, Investigation, Conceptualization. **Harald Kindermann:** Writing – review & editing, Software, Resources. **Kurosch Yazdi-Zorn:** Writing – review & editing, Validation, Resources. **Gerstgrasser Nikolas W.:** Writing – review & editing, Investigation, Conceptualization.

## Ethical approvals

The study was conducted ethically in accordance with the World Medical Association Declaration of Helsinki. The study protocol was approved by the local ethics committee on human research: JKU MED Ethics Committee, at the Kepler University Hospital Linz (Study No. 1171/2020). Written informed consent was obtained from all individuals to participate in the study.

## Declaration of Generative AI and AI-assisted technologies in the writing process

During the preparation of this work the authors used ChatGPT (OpenAI) in order to edit language and improve the flow of the text. After using this tool, the authors reviewed and edited the content as needed and take full responsibility for the content of the published article.

## Declaration of Competing Interest

The authors declare that they have no known competing financial interests or personal relationships that could have appeared to influence the work reported in this paper.

## Data Availability

Data and protocols are not publicly available to ensure special caution in handling patient information, but they can be provided upon reasonable request.
